# Origami rules for the construction of localized eigenstates of the Hubbard model in decorated lattices

**DOI:** 10.1038/srep16852

**Published:** 2015-11-19

**Authors:** R. G. Dias, J. D. Gouveia

**Affiliations:** 1 Department of Physics, I3N, University of Aveiro, Campus de Santiago, Portugal.

## Abstract

We present a method of construction of exact localized many-body eigenstates of the Hubbard model in decorated lattices, both for *U* = 0 and *U* → ∞. These states are localized in what concerns both hole and particle movement. The starting point of the method is the construction of a plaquette or a set of plaquettes with a higher symmetry than that of the whole lattice. Using a simple set of rules, the tight-binding localized state in such a plaquette can be divided, folded and unfolded to new plaquette geometries. This set of rules is also valid for the construction of a localized state for one hole in the *U* → ∞ limit of the same plaquette, assuming a spin configuration which is a uniform linear combination of all possible permutations of the set of spins in the plaquette.

The field of itinerant geometrically frustrated electronic systems has attracted considerable interest in the last two decades^1^,^2^,^3^,^4^,^5^,^6^,^7^,^8^,^9^,^10^,^11^,^12^,^13^,^14^,^15^,^16^,^17^,^18^,^19^,^20^,^21^,^22^,^23^,^24^,^25^,^26^. Much of this interest was related with the study of flat-band ferromagnetism in these systems^15^,^16^,^17^,^18^,^19^,^20^. Flat-band ferromagnetism occurs in decorated lattices of the Mielke’s and Tasaki’s classes, which display degenerate localized ground states with overlapping probability densities^21^,^22^,^23^,^24^,^25^,^26^. The emerging ferromagnetism can be interpreted as resulting from a generalized Hund’s rule^27^. In the case of the lattices which fall into the Lieb’s class, the flat bands intercalate itinerant bands^28^ and mean-field studies of the Hubbard Hamiltonian in the Lieb lattice indicate that for large *U*, ferromagnetism is expected except near half-filling where a ferrimagnetic phase appears^29^,^30^.

These localized states are one-particle eigenstates of the tight-binding Hamiltonians for the decorated lattices and little is known about the many-body eigenstates of an interacting system of fermions in decorated lattices^31^ (assuming Hubbard-like interactions), besides the appearance of a ferromagnetic ground state in decorated lattices of the Mielke’s and Tasaki’s classes^8^,^22^. In particular, the interacting ground state of the Hubbard model is not known in the case of lattices of the Lieb’s class. Approximate analytic results can be obtained in principle in the weak coupling limit, for example applying a recently proposed procedure that detangles the localized states from the dispersive states^32^,^33^,^34^, and introducing the Hubbard interaction as a perturbation of the tight-binding detangled lattice.

In this manuscript, we present a method of construction of exact localized many-body eigenstates of the Hubbard model in decorated lattices of arbitrary dimensions, for *U* = 0 and *U* → ∞. These states are localized in what concerns hole and particle movement. This method relies in simple arguments which lead to a set of quantum “origami” rules: i) if one plaquette or a set of plaquettes has a higher symmetry than that of the whole lattice, one can find energy eigenstates that have zero probability density at the sites that connect the plaquette or the set of plaquettes to the rest of the lattice (this argument is enough to justify the existence of localized states in the case of two-dimensional decorated lattices of the Lieb’s class); ii) given such a localized state in the symmetric plaquette, one can fold the plaquette, either at the probability density nodes or at other equivalent sites (adjusting the probability density at those sites and the hopping constants that involve those sites), therefore lowering the symmetry of the plaquette; iii) the energy of the localized state can be lowered by adding hopping terms between sites with the same localized state phase (if the hopping constant is negative) or hopping terms between sites with opposite phases (if the hopping constant is positive). Hopping terms between nodes of the localized state may also be added, but do not change the energy of the localized state. The hopping terms added must preserve the symmetry of the localized state. These two arguments justify localized states in decorated lattices of the Mielke’s and Tasaki’s classes. Furthermore, the spin degree of freedom of the *U* = 0 Hubbard Hamiltonian may be interpreted as a sublattice index and localized states can also be created using these origami rules involving the two (up and down spin) sublattices. Such localized states arise for instance as edge states in 1D tight-binding descriptions of topological insulators^35^,^36^,^37^.

The remaining part of this paper is organized in the following way. First, we review the construction of one-particle localized eigenstates of the tight-binding decorated lattices of the Lieb’s class. We then generalize this construction to more complex lattices using a symmetry argument and introducing the set of origami rules. Next, we show how to extend these rules to the case of the *U* → ∞ limit of the Hubbard model. Finally we conclude.

The Hubbard Hamiltonian in a decorated lattice can be written as


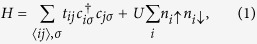


where the creation (annihilation) of an electron at site *i* with spin *σ* is denoted by 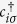 (*c*_*iσ*_) with *n*_*iσ*_ being the number operator 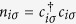 and *n*_*i*_ = *n*_*i*↑_ + *n*_*i*↓_. The sum over 〈*ij*〉 is the sum over all pairs of sites with a finite hopping probability between them and this a different sum for each decorated lattice. The hopping constants are assumed to be equal, *t*_*ij*_ = *t*, unless stated otherwise. When *t* = 0, all states with the same number *N*_*d*_ of doubly occupied sites are degenerate. In this paper, we assume *N*_*d*_ = 0. The Hubbard model in the limit *U* → ∞ is also designated as Harris-Lange model^38^. In this limit, using the identity *c*_*iσ*_ = *c*_*iσ*_[(1 − *n*_*iσ*_) + *n*_*iσ*_], the Hubbard model can be rewritten as


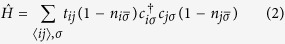


with 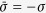. An important point about the strong coupling limit is that the Hamiltonian eigenfunctions, in the case of a Hubbard ring, can be written as a tensorial product of the eigenfunctions of a tight-binding model of independent spinless fermions (holes) in the ring with *L* sites and the eigenfunctions of an Heisenberg model (with exchange constant *J* = *t*^2^/*U*) in a reduced chain^39^,^40^,^41^,^42^.

## Origami rules for tight-binding Hamiltonians

Let us first discuss the *U* = 0 case of the Hubbard model in decorated lattices. Flat bands in the one-particle tight-binding energy dispersion of geometrically frustrated lattices reflect the existence of degenerate localized eigenstates which are translated versions of the same state 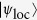. The probability density associated with one of these localized states is non-zero only in a small lattice region. In the particular case of decorated lattices of the Lieb’s class, the localized states can be viewed as one-dimensional standing waves in tight-binding rings, associated with paths in the 2D lattice which include one or two plaquettes^43^. For zero flux, all one-particle energy levels of a tight-binding ring (with even number of sites) are doubly degenerate (except for *k* = 0 and *k* = *π*) and the respective eigenstates have opposite momenta. Adding or subtracting the states of opposite momenta, one obtains a standing wave with a number of nodes that depends on *k*. If these nodes coincide with the sites at the vertices of a plaquette of a decorated lattice, the electron becomes trapped inside the plaquette. Therefore, flat band eigenstates of decorated lattices of the Lieb’s class are constructed from standing waves such that the nodes coincide with sites at the vertices. Note that these localized states overlap in real space, that is, they constitute a basis of the subspace of localized states but not an orthogonal basis.

**Figure 1:**
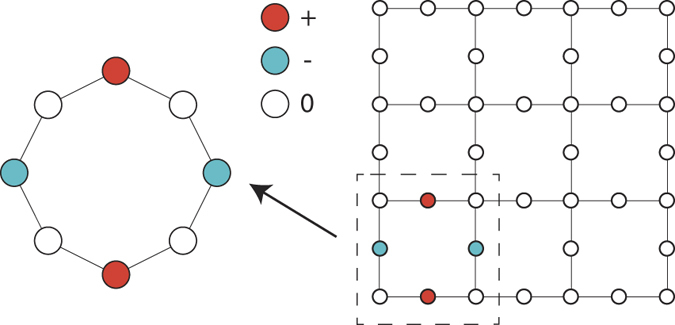
The symmetry of the tight-binding Hamiltonian for the Lieb plaquette is the same as that of the tight-binding ring and larger than that of the Lieb lattice. The distance between adjacent sites is assumed to be *a* = 1. All hopping constants are equal.

**Figure 2:**
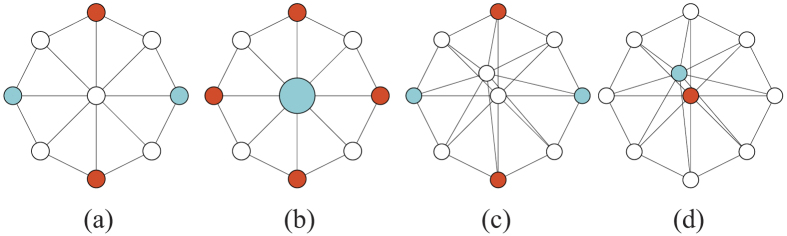
In these simple variations that retain the symmetry of the Lieb plaquette, two localized states exist corresponding to two possible choices of angular momentum. The size of the circles that represent lattice sites indicates the relative value of the wavefunction amplitudes. There is an analogy of these states with atomic orbitals.

The previous argument for lattices for the Lieb’s class can be generalized to decorated lattices of the Mielke’s and Tasaki’s classes and other decorated lattices using a symmetry argument. First, let us discuss the case of the Lieb lattice (see Fig. 1). The tight-binding Hamiltonian of one plaquette of the Lieb lattice has the symmetry of a ring of 8 sites, that is, the plaquette Hamiltonian is invariant in a 2*π*/8 rotation of the set of site indices (or equivalently in a circular permutation of the set of site indices). We emphasize that this rotation should not be confused with a 2*π*/8 rotation in real space (the plaquette is not invariant in such a rotation). However, the rotation of 2*π*/4 in the set of sites indices can be interpreted as a 2*π*/4 rotation in real space. In the ring of 8 sites, one has outer sites (that are connected to the rest of the lattice) and inner sites (with connections only to sites of the plaquette). In the case of Fig. 1, the inner sites are indicated by the filled circles and the outer sites are given by the empty circles. The generator of this rotation symmetry is the equivalent of the angular momentum in the ring (note that in a 2D lattice, the direction of the angular momentum is always perpendicular to the lattice and therefore equal to *mħ*, where *m* can be interpreted as the *m* in the ring momentum *k* = *m* ⋅ 2*π*/*N*) and one can construct an eigenbasis of the Hamiltonian which is simultaneously an eigenbasis of the angular momentum. The time reversal symmetry of the Hamiltonian implies that each eigenstate of the plaquette tight-binding Hamiltonian is degenerate with the respective state obtained in a time reversal and these states have opposite angular momenta (this is equivalent to stating that ring eigenstates with momenta *k* and −*k* are degenerate). These two states can be added or subtracted, generating the equivalent of the standing waves in the ring, that is, states with zero probability density at certain sites of the cluster. If the angular momentum is *ħN*/4, where *N* is the number of sites of the ring, one has zero probability density at the inner sites or at the outer sites of the Lieb plaquette (nodes are separated by *λ*/2 = 2). The latter will be a localized eigenstate not only of the Lieb plaquette but also of the tight-binding Hamiltonian of the full lattice. Note that this description is valid for any plaquettes which have the same rotation symmetry as the ring. For example, one could add additional sites at the center of the Lieb plaquette and the rotation symmetry would remain, as shown in Fig. 2 (in all Figures, the relative size of the circles that represent lattice sites corresponds to the relative value of the wavefunction amplitudes on the sites).

**Figure 3:**
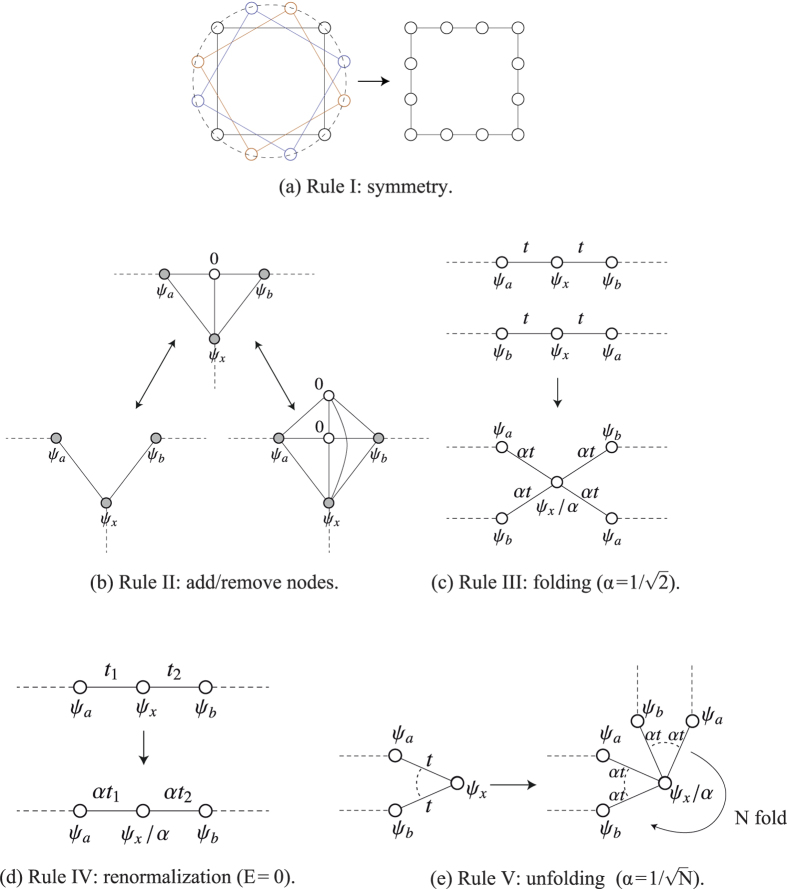
Set of origami rules for the construction of localized states in decorated lattices. The values of *α* can be obtained from simple tight-binding calculations, imposing the condition that the state is still an eigenstate after applying the rule.

Thus, our first rule is that localized states can be constructed if a plaquette (or a set of adjacent plaquettes) has a larger symmetry than the lattice, so that the Hamiltonian has two degenerate eigenstates (which are simultaneously eigenstates of the generators of the symmetry of the lattice) which have the same wavefunction values at the outer sites of the plaquette (see Fig. 3a). This rule is enough to explain the existence of localized states in lattices of the Lieb’s class. More complex lattices with localized states can be constructed by adding sites or hopping bonds that do not lower the symmetry of the plaquette. These additional hoppings can be divided into two sets: i) the set of hoppings from or to sites with probability density nodes (these hoppings do not modify the energy of the localized state); ii) the set of hoppings between sites with finite density probability (these hoppings lower or raise the energy of the localized state).

**Figure 4:**
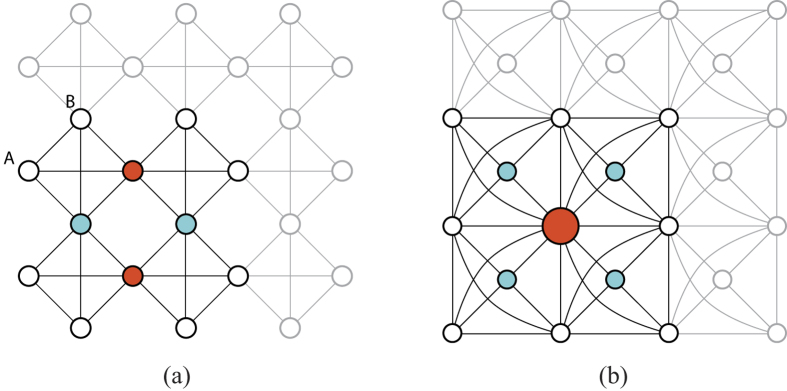
Localized states in the (a) Mielke lattice and (b) Tasaki lattice. All hopping constants are the same (*t*_*ij*_ = *t*), except those associated with curvy lines (*t*_*ij*_ = *t*/2). Note that the tight-binding Hamiltonian for the Mielke plaquette is symmetric in the exchange of sites A and B, but the full tight-binding Hamiltonian is not. This implies the Hamiltonian eigenfunctions must have the same amplitude value (or opposite values) at sites A and B. In the case of the localized states, the value must be the same and the sites A and B are effectively one site.

A second rule for the construction of lattices with localized states is the following. The existence of sites where a localized state has probability density nodes does not affect the energy of the state and these sites can be dropped, duplicated (as well as the respective hopping bonds), or simply added (introducing appropriate hoppings with neighboring sites) and the localized state remains an eigenstate of the modified tight-binding model associated with the new plaquette geometry (see Fig. 3b). Furthermore, if one can draw an axis through the plaquette that crosses only nodes, then dropping these nodes one divides the localized state into two eigenstates of the tight-binding Hamiltonians associated with the parts of the plaquette. Bonds between nodes can also be dropped, added or duplicated. This rule justifies the localized states in the lattice of Fig. 4a. In fact, sites A and B in Fig. 4a can be seen as a duplication of the equivalent site of the ring of Fig. 1, with the addition of a hopping bond between the duplicated nodes.

The third rule consists of the following: localized states can be folded (adjusting the amplitude at the crossing and the respective hoppings) along an axis that crosses the plaquette through sites that have the same wavefunction values (see Fig. 3c); if the folding is along an axis that crosses nodes, no adjustment of hopping constants or wavefunctions amplitudes is needed.

The fourth rule is that the amplitude at a given site of a localized state with zero energy can be renormalized without changing the energy of the state, if the hopping constants to that site are renormalized as well (see Fig. 3d).

The fifth rule describes the unfolding of a plaquette around a given site (see Fig. 3e). Multiple rotated copies of the original plaquette can be added around a site, provided that the amplitude of the wavefunction on this site is adjusted, as well as the hopping constants around this site.

**Figure 5:**
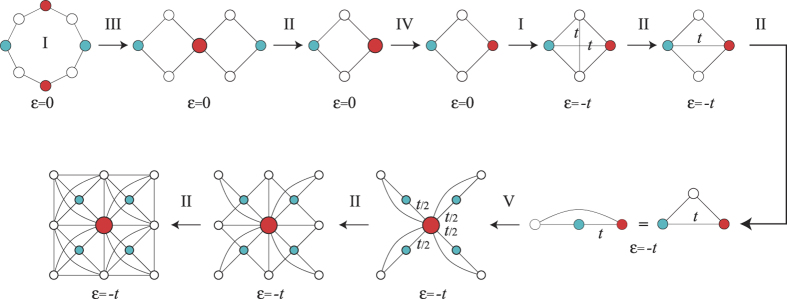
Application of the rules presented in the text, starting from the localized state in the Lieb plaquette and ending at the localized state in the Tasaki lattice. Note that the energy of the localized state in the Tasaki lattice is determined by the central hoppings between sites with finite probability density.

This set of rules justifies the existence of localized states in the Mielke and Tasaki lattices of Fig. 4. In Fig. 5, we exemplify the application of this set of rules starting from the localized state of the Lieb plaquette and ending at the localized state of the Tasaki lattice.

We emphasize that these rules can be applied to construct localized states in systems of arbitrary dimension, from 0D (a molecule) to 3D crystals, since the tight-binding bonds of Fig. 3 may not be coplanar and the unfolding axis can have an arbitrary direction. Furthermore, the spin degree of freedom of a tight-binding model may be interpreted as a sublattice index, and spin flipping terms can be interpreted as hopping terms between such sublattices. Localized states can also be created using the above origami rules involving the two (up and down spin) sublattices and examples of such localized states are the edge states in 1D models of topological insulators with spin flipping terms (see for example section 3.3. of ref. 35).

Another context where this set of rules applies is that of one-magnon localized states in frustrated quantum Heisenberg antiferromagnets. In these systems, these states generate peculiar behavior such as magnetization plateaus around the saturation field^15^,^44^.

## Origami rules in the *U* → ∞ limit of the Hubbard model

Let us now discuss how much of this method can be applied in the *U* → ∞ limit of the Hubbard model. We start by considering the Lieb lattice and then we generalize our conclusions to more complex decorated lattices. As discussed previously, the Lieb plaquette is a 8-site tight-binding ring. The eigenfunctions of the Hubbard ring in the strong coupling limit have been obtained by several authors^39^,^40^,^41^,^42^. Here, we present a simple derivation of the one-hole case when no doubly occupied sites are present. Our approach follows closely the method and notation used by Dias and Peres^41^,^42^.

Let us consider a state of the *U* → ∞ Hubbard ring with *N* sites and a single hole at site *i*, given by


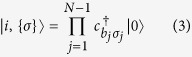


where {*σ*} = {*σ*_1_,…, *σ*_N−1_} is the set of particle spins in the lattice and *b*_*j*_ = *j* if *j* < *i* and *b*_*j*_ = *j* + 1 if *j* ≥ *i*. Now we introduce an operator 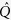 such that


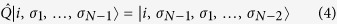


that is, it does a circular permutation of the spin configuration. When the Hamiltonian given by Eq. 2 acts on such a state it simply exchanges the empty site with a spin with amplitude *t* without changing the spin sequence {*σ*}, except when the hole is at sites 1 and *L*. If the hole hops from site 1 to site *N*, a new spin sequence is obtained which is the circular permutation of the previous one, 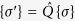. Therefore the hole motion mixes the spin configurations with those spin configurations which are circular permutations of the initial one.

Given a configuration of spins {*σ*}, the eigenstates of the Hamiltonian will be found in the subspace spanned by 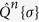, 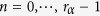, where *r*_*α*_ is the period of the spin configuration. The *α* index labels the different (not obtainable from any other by cyclic permutations) spin configurations with period *r*_*α*_. Let us build states invariant in a circular permutation


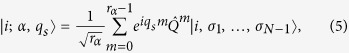


where *q*_*s*_ = *n*_*s*_(2*π*/*r*_*α*_) with 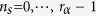, that is, a state such that


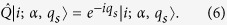


Since the hoppings across the boundary do a cyclic permutation, the Hamiltonian in the subspace of states 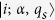 becomes


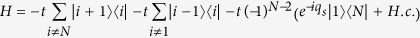


where we have simplified the notation by dropping the spin configuration index *α* and the spin momentum *q*_*s*_. This is a simple tight-binding model of one spinless fermion (hole) with twisted boundary conditions and the respective eigenstates are Bloch states for the movement of the hole. A gauge transformation 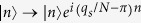 makes the previous model translation invariant and one has


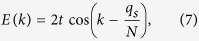


with *k* = (2*π*/*N*)*n*, *n* = 0,…, *N* − 1.

In the case of this paper, the one-hole solution in the *U* → ∞ limit of the Hubbard ring is sufficient, but note that this solution can be generalised to the case of *N*_*h*_ holes in the Hubbard ring (in the *t*^2^/*U* ≪ 1 limit) with the Hubbard ring eigenfunctions being written as a tensor product of the eigenfunctions of a tight-binding model of independent spinless fermions (holes) in the ring with *L* sites and the eigenfunctions of an Heisenberg model (with exchange constant *J* = *t*^2^/*U*) in a reduced chain^39^,^40^,^41^,^42^.

**Figure 6:**
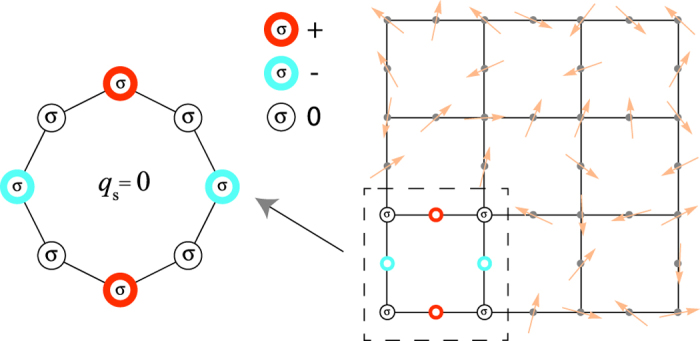
One hole can be trapped in a plaquette of the half-filled interacting Lieb lattice, in the *U* → ∞ limit of the Hubbard model, if the spin momentum of the spin configuration in that plaquette is zero. Since the hole probability density is zero at the outer sites of the plaquette, this plaquette effectively decouples from the rest of the lattice, where the spin configuration is arbitrary. The red and blue sites indicate finite amplitude of the hole wavefunction (positive and negative, respectively).

So in the case of one hole, the energy dispersion given by Eq. 7 is that of one spinless fermion in a tight-binding ring threaded by a fictitious magnetic flux, *ϕ* = *q*_*s*_, generated by the spin configurations in the reduced Heisenberg chain (where *q*_*s*_ is the spin momentum of the chain). Therefore, if the spin momentum is zero (note that non-zero spin momentum destroys the time reversal symmetry of the tight-binding model of spinless fermions), one can construct a standing wave for one hole moving in the Hubbard ring with arbitrary spin configuration as in the case of one particle in a tight-binding ring shown in Fig. 1. Since the Lieb plaquette is a ring with 8 sites, this standing wave for one hole can be created in this plaquette with nodes of the hole probability density at the outer sites as shown in Fig. 6 (this standing wave is obtained combining the degenerate states with *k* = *π*/2 and *k* = −*π*/2). Since the hole is trapped in a plaquette (indicated by the dashed square in Fig. 6), the spin configuration in the rest of the lattice is arbitrary, in the limit of the Harris-Lange model. We emphasize that the spin momentum mentioned above is that of the spin configuration in the Lieb plaquette (a ring of 8 sites) and not that of the spin configuration of the total lattice (we designate the set of sites of the Lieb lattice as Λ). More precisely, the localized hole state in the Lieb lattice will be the antisymmetric tensor product of the hole state in the Lieb plaquette and the state of the spins in the rest of the lattice (see Fig. 6). As usual, writing the full state using creation operators assures the proper antisymmetrization of the hole localized state in the Lieb lattice and if the hole is localized in a Lieb plaquette that we label as *i*_*p*_, this state has the form:


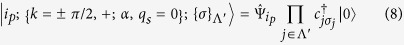


with


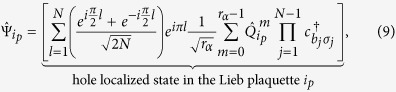


and where *N* = 8 is the number of sites in the Lieb plaquette (which are labelled clockwise from 1 to *N*, starting at an outer site), Λ′ is the set of sites of the Lieb lattice, excluding those of the plaquette where the hole is trapped (see dashed square in Fig. 6), *i*_*p*_ labels the plaquette in the Lieb lattice where the localized hole is, {*σ*}_Λ′_ is the spin configuration at the sites in Λ′, *l* indicates the position of the hole in the Lieb plaquette *i*_*p*_ and with *b*_*j*_ = *j* if *j* < *l* and *b*_*j*_ = *j* + 1 if *j* ≥ *l*. The operator 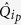 does a circular permutation of the spin configuration in a single Lieb plaquette (labelled *i*_*p*_). The standing wave is created summing the degenerate states (in the Lieb plaquette) with *k* = *π*/2 and *k* = −*π*/2 and this is indicated by {*k* = ± *π*/2, +} in Eq. 8.

This construction can be generalized to an arbitrary number of localized holes, each one of them in a different plaquette, as long as the plaquettes are not contiguous (plaquettes which do not have common sites),


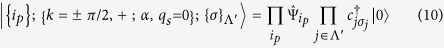


with Λ′ being the set of sites of the Lieb lattice remaining after dropping the sites associated with the plaquettes {*i*_*p*_}. The sites in each of these plaquettes are labelled using two indices, (*i*_*p*_, *l*) with *l* = 1, …, *N*, so that the hole localized states in each of these plaquettes have still the form given by Eq. 9 with *b*_*j*_ → (*i*_*p*_, *b*_*j*_).

Does this apply to more complex plaquettes that share the rotation symmetry of the Lieb plaquette? Taking the example shown in Fig. 2a, one sees that in the non-interacting case, a localized state is present where the particle is confined to a 1D path. This leads one to suggest that an equivalent localized state can be constructed for the hole moving in the spin background, if we impose a *q*_*s*_ = 0 spin momentum for the spins configuration in the 1D path (note that we are using the same definition of spin momentum as above, since the 1D path in Fig. 2a correspond to that of the Lieb plaquette). However one should note that in Fig. 2a, despite the electron probability density being finite only in the outer ring, when the electron is at the outer ring, it still hops to the center site, but summing over all the hopping possibilities from the sites at the outer ring to the center site, the result will be zero (destructive interference). In the case of the hole moving in the spin background, the hops of the hole from sites at the outer ring to the center site mix the spins at the outer ring and at the center. In order for one to have destructive interference at the center, the spin configuration must be a uniform linear combination of all possible permutations of the set of spins (given the number of up spins and down spins). The reason is the following: when an electron in the localized state of Fig. 2a hops from a site of the outer ring to the center site, it interferes destructively with the contributions of hoppings from the other sites of the outer ring. In the case of the hole, one has the different spin backgrounds and the hops of the hole from the outer ring to the center should not apparently interfere destructively. However, if one works with hole states such that, for a given number of up and down spins in the plaquette, the spin configuration is a uniform linear combination of all possible permutations of the set of spins, then: i) the spin configuration in the outer ring has *q*_*s*_ = 0 spin momentum, that is, one can apply circular permutations to the spin configuration in the outer ring but this will not modify the spin configuration; ii) a hole jump from a site A or B of the outer ring to the central site will generate the same final state, independently of the initial site being A or B. Therefore, we have the same localized state for one hole in the *U* → ∞ limit of the Hubbard model as for one electron when *U* = 0. Or better, the spin configuration in the background will be equivalent to a saturated ferromagnetic configuration in what concerns the movement of the hole, since the spin configuration will not change and will not generate additional phase factors. This state can be expressed as 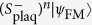, where 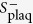 is the spin-lowering operator defined within the plaquette, *n* is the number of down spins and 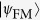 is the plaquette ferromagnetic state with one hole, which can be written as 
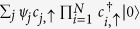
, where *ψ*_*j*_ is the hole wavefunction amplitude, where j labels all sites of the plaquette where the hole is localized.

**Figure 7:**
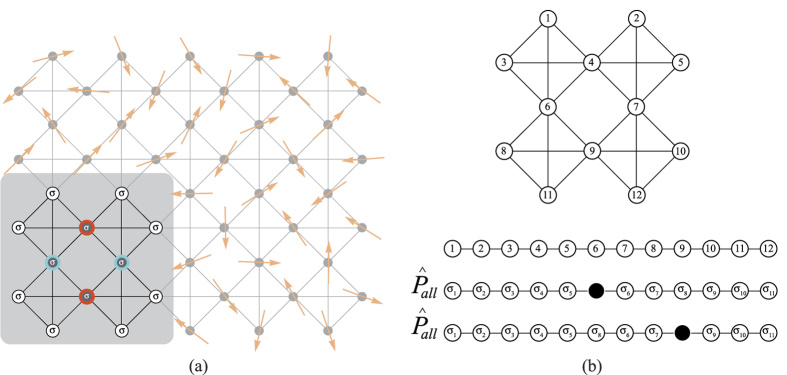
(**a**) The construction of a localized state for one hole in the *U* → ∞ limit in an arbitrary decorated lattice is possible (if one-particle localized states exist), assuming a spin configuration which is a uniform linear combination of all possible permutations of the set of spins in the plaquette or set of plaquettes associated with the compact one-particle localized states (gray region). (**b**) Top: Numbering of sites in the set of plaquettes where the hole localized state has finite probability density; bottom: spin configuration using a chain representation of the set of plaquettes, in the case of one hole at site 6 and one hole at site 9, after a 6 → 9 hopping.

Let us explain in more detail the last argument using the example of the Mielke lattice in Fig. 7a. The localized hole will have finite probability in the same compact set of plaquettes as the compact localized state of the tight-binding limit (see Fig. 4a). If we number the sites in this compact set of plaquettes as shown in Fig. 7b, one can use a mathematical formalism similar to the one used above in the case of the interacting Lieb lattice. When one has a hole at site 6 and the hole hops to site 9, this hopping induces a modification of the spin configuration from {*σ*} = {*σ*_1_,…, *σ*_11_} to {*σ*′} = {*σ*_1_, *σ*_2_, *σ*_3_, *σ*_4_, *σ*_5_, *σ*_8_, *σ*_6_, *σ*_7_, *σ*_9_, *σ*_10_, *σ*_11_} (see Fig. 7b). Let us introduce the operator 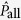 that sums over all different permutations {*σ*′} of the spin configuration {*σ*} (note that a permutation of two up-spins does not lead to a different spin configuration),


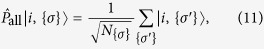


where *N*_{*σ*}_ is the number of different permutations of the spin configuration {*σ*}. The construction of a state involving the sum over spin permutations resembles the construction of the ground state in the Brandt-Giesekus model^45^,^46^, although in this model only a particular filling is considered. Starting from the state 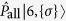, the hopping of the hole from site 6 to site 9 leads to 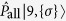, that is, the spin configuration does not change (note that {*σ*′} is a permutation of {*σ*}, so the sum of all permutations of {*σ*′} is equal to the sum of all permutations of {*σ*}). Another way to state this is the following: any operation of transposition of two spins commutes with 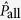, and therefore any permutation of the spin configuration {*σ*} commutes with 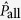. Therefore, if 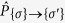 is the permutation of {*σ*} into {*σ*′}, then 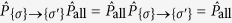. Note that an extra minus sign could appear if the hopping of the hole involves an odd number of exchanges of creation operators, but this extra sign is the same as in the case of the same hopping of the hole in a saturated ferromagnetic background (assuming the same numbering of sites). Since it is obvious that we can create a hole localized state in a decorated lattice with a saturated ferromagnetic background (one can do a particle-hole transformation and the hole localized state becomes a particle localized state), this implies that a hole localized state can be created in the set of plaquettes shown in the gray region of Fig. 7a. The spin configuration in the rest of the lattice is arbitrary.

This construction of a localized state is valid for any decorated lattice where a localized state of one tight-binding electron exists. In the case of the states given by Eq. 10, the localized holes occupy plaquettes with no common sites (which limits the maximum number of localized holes to a fourth of the number of plaquettes, in the case of the Lieb lattice). Note however that in the case of the plaquette with ferromagnetic background (or equivalently plaquette states with a spin configuration which is a uniform linear combination of all possible permutations of the plaquette set of spins), the maximum number of localized holes is higher since the ferromagnetic configuration can be shared by plaquettes that have common outer sites (sites with nodes of the hole wavefunction) but no common inner sites (in the Lieb lattice, this would lead to a maximum of localized holes equal to half the number of plaquettes). This has been confirmed numerically, diagonalizing exactly the Harris-Lange model in a set of few plaquettes for several choices of decorated lattices.

Note that besides the localized state degeneracy associated with the choice of the lattice plaquette where the localized state sits, there is a huge degeneracy associated with the possible choices of number of up spins (or down spins) in the plaquette and in the rest of the lattice. This degeneracy is lifted by the Heisenberg corrections of order *t*^2^/*U*, as in the Hubbard ring^39^,^40^,^41^,^42^.

Another important remark is that while the one-electron localized states of the Mielke and Tasaki lattices are the ground states of the respective tight-binding Hamiltonians, in the case of the *U* → ∞ Hubbard model, the one-hole localized state is not the ground state, for the choice of relative hopping constants of Fig. 4. However, since the exact form of the hole probability density is known as well as the hole wavefunction phase (as in the case of the one-electron localized state), it is possible to tune the geometry, hopping constants and interactions in order to lower the energy of the hole localized state relatively to the other states, so that the energy of the localized state approaches the energy of the ground state.

## Conclusion

In conclusion, we have presented a simple set of rules for the construction of localized states of the Hubbard model in nearly arbitrary decorated geometries, in the tight-binding limit (*U* = 0), and in the strong-coupling limit (*U* → ∞). The first step in this method is the choice of a plaquette or a set of plaquettes with a higher symmetry than that of the whole lattice. In this plaquette, one has a localized state of the tight-binding Hamiltonian of the full lattice (this state has probability density nodes at the sites shared between the plaquette and the rest of the lattice). Using a simple set of rules, the tight-binding localized state in such plaquette can be divided, folded or unfolded to new plaquette geometries. We have shown that this set of rules can also be applied in the *U* → ∞ limit of the Hubbard model, for the construction of localized states of one hole in the plaquette, assuming a spin configuration which is a uniform linear combination of all possible permutations of the set of spins in the plaquette. Note that in every other plaquette, one may place a localized hole, so localized hole states exist for hole doping between zero and a value of the order of 1/*N*_loc_ (the value depends on the lattice geometry), where *N*_loc_ is the number of atomic sites of the set of plaquettes where one hole is localized.

This paper presents a unifying picture of construction of localized states, in tight-binding systems of arbitrary dimension (from 0D to 3D), arbitrary geometry (including Mielke’s and Tasaki’s 2D geometries), without and with interactions (*U* = 0 or *U* = ∞, extending in the latter case the filling intervals where localized states are known to occur). The existence of localized states due to spin flipping terms in tight-binding descriptions of topological models, or the existence of one-magnon localized states in frustrated Heisenberg antiferromagnets, are two other contexts included in this unifying picture.

## Additional Information

**How to cite this article**: Dias, R. G. and Gouveia, J. D. Origami rules for the construction of localized eigenstates of the Hubbard model in decorated lattices. *Sci. Rep.* **5**, 16852; doi: 10.1038/srep16852 (2015).
